# Age and processing effects on perceptual and conceptual priming

**DOI:** 10.1177/17470218221090128

**Published:** 2022-04-03

**Authors:** Emma V Ward

**Affiliations:** Faculty of Science and Technology, Psychology Department, Middlesex University, London, UK

**Keywords:** Ageing, processing, implicit memory, perceptual priming, conceptual priming

## Abstract

Explicit (declarative) memory declines with age, but age effects on implicit (nondeclarative) memory are debated. Some studies have reported null changes in implicit memory (e.g., priming in word-fragment completion, perceptual identification, category exemplar generation) with age, while others have uncovered declines. One factor that may account for these discrepancies is processing. Evidence suggests that conceptual and perceptual processes are not equally affected by ageing, yet processing requirements have varied greatly between studies. Processing may moderate age effects on priming, but no study has systematically examined this issue. This registered report presents an experiment to manipulate processing (conceptual / perceptual) during incidental encoding of words, prior to measures of perceptual (perceptual identification) and conceptual (category verification) priming. The perceptual and conceptual priming tasks were matched on all characteristics except processing, making them highly comparable. The four orthogonal conditions (perceptual encoding, perceptual test [PP]; conceptual encoding, perceptual test [CP]; perceptual encoding, conceptual test [PC]; conceptual encoding, conceptual test [CC]) were designed to clarify situations in which age effects on implicit memory emerge, which holds important practical and theoretical implications. Significant effects of Age, Test, and an Age × Processing interaction emerged. Priming was greater in young than older adults and on the perceptual than the conceptual test, but in contrast to the predictions, the age difference was only significant when prior encoding was perceptual (i.e., in the PP and CP conditions).

Understanding age-related changes in memory is increasingly important in light of the steady rise in age of the global population. Reductions in explicit memory—the conscious retrieval of previously learned information or prior experiences—are well documented, but age effects on implicit memory—facilitation in task performance due to prior exposure to stimuli which does not require conscious recollection—are debated. If, as it is commonly believed, implicit memory is preserved in normal ageing, this could have important practical and theoretical implications. For example, it might provide a valuable diagnostic tool for Alzheimer’s disease (Fleischman, 2007) or be used to aid everyday tasks such as learning medication routines or new face–name associations (Haslam et al., 2011). Moreover, spared implicit memory in the face of explicit decline is often taken as evidence for functionally independent memory systems and has shaped theory (e.g., Gabrieli, 1998; Schacter & Tulving, 1994; Squire, 2004). Unfortunately, the literature is replete with contradictory findings.

A number of studies have reported statistically equivalent priming in young and older adults on tests such as word-stem completion (WSC; e.g., Jelicic et al., 1996; Light & Singh, 1987; Mitchell & Bruss, 2003; Park & Shaw, 1992; Spaan & Raaijmarkers, 2010), word-fragment completion (e.g., Light et al., 1986; Mitchell & Bruss, 2003), perceptual identification (e.g., Henson et al., 2016; Light et al., 1992; Light & Singh, 1987; Sullivan et al., 1995; Wiggs et al., 2006), picture naming (e.g., Mitchell, 1989; Mitchell & Bruss, 2003; Mitchell et al., 1990; Mitchell & Schmitt, 2006), lexical decision (e.g., Karavanidis et al., 1993; Moscovitch, 1982), homophone spelling (e.g., Howard, 1988), and category exemplar generation (CEG; e.g., Isingrini et al., 1995; Light et al., 2000; Mitchell & Bruss, 2003). Such observations have been taken as a key strand of evidence for multiple memory systems (e.g., Gabrieli, 1998, 1999; Mitchell, 1989; Mitchell et al., 1990; Schacter, 1987; Schacter & Tulving, 1994; Squire, 1994, 2004, 2009; Tulving & Schacter, 1990). The argument is that selective deficit to explicit memory function with age, coupled with preserved implicit memory, is expected if the two are driven by functionally independent systems. However, it has also been argued that single dissociations (i.e., reliable age effects on explicit memory coupled with a nonsignificant differences in priming) do not constitute strong evidence for independent systems because age effects on priming may often go undetected (e.g., Buchner & Wippich, 2000; Ward et al., 2013b; Ward & Shanks, 2018).

Reduced priming in older compared with young adults has been reported on tests of WSC (e.g., Chiarello & Hoyer, 1988; Davis et al., 1990; Small et al., 1995), unfamiliar word/object naming (e.g., Keane et al., 2004; Soldan et al., 2009; Wiggs & Martin, 1994), CEG (Stuart et al., 2006), category verification (CV; Light et al., 2000), perceptual identification (e.g., Abbenhuis et al., 1990; Russo & Parkin, 1993; Ward, 2018; Ward et al., 2013a, 2013b, 2020), and homophone spelling (e.g., Davis et al., 1990; Howard, 1988; Rose et al., 1986). These observations are consistent with the view that explicit memory and implicit memory are driven by a single underlying system (e.g., Berry et al., 2006, 2008a, 2008b, 2012; Buchner & Wippich, 2000; Nosofsky et al., 2012). On the whole, cognitive ageing provides a fruitful platform from which to investigate issues around the organisation of memory, but given the volume of mixed findings there is currently no clear answer to the question of whether ageing affects implicit memory.

One likely contributor to the discrepancies is processing characteristics (for in-depth reviews, see Fleischman, 2007; Mitchell & Bruss, 2003; Ward & Shanks, 2018). Age effects may differ between studies depending on the specific cognitive processes that are engaged. A broad distinction has been made between perceptual and conceptual priming tasks (e.g., Roediger & Blaxton, 1987). In perceptual tasks, stimuli from the encoding phase are represented at test, sometimes in a degraded form. In the case of perceptual identification, participants are required to identify words/objects as quickly as possible, and priming is evidenced by faster identifications of previously presented than new items. Conceptual tasks, on the other hand, draw upon semantic processing of primed items (i.e., content and meaning). For example, in CEG, participants spontaneously produce words related to a category cue (e.g., items of clothing), and priming occurs if more previously studied than new words are produced. Other perceptual tasks include word-fragment completion, lexical decision, and anagram solving, and other conceptual tasks include word association, category verification (CV), and fact completion. A key distinguishing feature of perceptual and conceptual priming tasks is that perceptual tasks are sensitive to physical changes in stimuli between study and test (e.g., modality changes) while conceptual tests are not, and conceptual tests are sensitive to elaborative processing manipulations (e.g., levels-of-processing) while perceptual tests are not (for reviews, see Mulligan, 2004; Roediger & McDermott, 1993).

The ability to engage in conceptual processing declines to a greater extent with age than perceptual processing (e.g., Rybash, 1996), and there is also evidence that the capacity to process semantic information is correlated with higher fluid intelligence in older adults, while perceptual processing is unrelated to such cognitive measures (e.g., Bruffaerts et al., 2019). This leads to the prediction that age effects will be larger on conceptual priming tasks than on perceptual ones (see Geraci & Hamilton, 2009; Roediger & Blaxton, 1987a, 1987b; Weldon, 1991). However, evidence that ageing selectively diminishes conceptual priming is mixed. Several studies have reported reliable age effects on conceptually driven tests including CEG (Jelicic et al., 1996; Maki et al., 1999; Maki & Knopman, 1996), word association (Grober et al., 1992), and CV (Light et al., 2000), but others have reported age-invariant priming in fact completion, word association, and CEG (e.g., Brooks et al., 2001; Isingrini et al., 1995; Java, 1996; Light & Albertson, 1989; Light et al., 2000; McEvoy et al., 1995; Mitchell & Bruss, 2003; Small et al., 1995). Furthermore, as reviewed above, a range of studies have reported reduced priming in older adults on perceptual tasks (e.g., Abbenhuis et al., 1990; Chiarello & Hoyer, 1988; Keane et al., 2004; Russo & Parkin, 1993; Soldan et al., 2009; Ward et al., 2013b, 2020; Wiggs & Martin, 1994), and Small et al. (1995) uncovered a reliable age effect in perceptual but not conceptual priming within a single study.

To add to the ambiguity, processing at encoding has also varied across studies, with some encouraging conceptual encoding (e.g., liking or preference judgements, semantic categorisation), some encouraging perceptual encoding (e.g., letter counting, orientation judgements), and others requiring no particular strategy (i.e., simply presenting stimuli to participants). In the latter situation, it is impossible to know what sort of processing strategy was adopted by participants or whether this differed between young and older adults. There is extensive evidence that older adults are impaired in semantic encoding (e.g., Eysenck, 1974; Morcom et al., 2003; Morcom & Rugg, 2004), so one may expect age effects in priming to be greater in studies involving conceptual encoding and smaller/absent in studies involving perceptual encoding. There is some evidence for this when looking at prior studies (i.e., examples of significant age effects in studies that used conceptual encoding: Light et al., 2000; Russo & Parkin, 1993; Ward et al., 2013b; examples of null age effects in studies that used perceptual encoding: Park & Shaw, 1992; Soldan et al., 2009, Experiment 3), but considerable ambiguity remains as outcomes may also vary as a function of interactions between processing at encoding and test.

Stuart et al. (2006) manipulated processing during encoding by requiring participants to count vowels (perceptual) or make preference judgements (conceptual). Participants who performed perceptual encoding completed a perceptual priming task (WSC), and those who performed conceptual encoding completed a conceptual priming task (CEG). Priming was significantly reduced by age in the conceptual but not the perceptual condition. However, there was no examination of conceptual priming following perceptual encoding, or vice versa. In a more recent study, Ward et al. (2020) manipulated processing in a lifespan sample of over 1,000 participants aged 12–85 years, who performed either a conceptual (semantic categorisation) or a perceptual (angular/rounded judgement) encoding task prior to measures of perceptual priming and recognition. Age predicted significant reductions in both priming and recognition, but there was no interaction with processing. However, the authors concluded that the processing manipulation was ineffective, and no measure of conceptual priming was included. This presents an important gap in the literature—no study has crossed perceptual/conceptual processing at encoding and test to examine the effect on priming and interactions with age.

## Current investigation

Age effects on priming remain unclear. The varied outcomes among published studies may be explained by different processing requirements. Age differences may be small or statistically null when the priming task is perceptual in nature and encoding engages participants with perceptual features of the stimuli, and larger when the priming task is conceptual and encoding engages participants with semantic features of the stimuli. To clarify this issue, this registered report presents an experiment to systematically manipulate processing at encoding in young and older adults prior to matched perceptual and conceptual priming tests.

Participants witnessed a stream of words in two counterbalanced incidental encoding blocks, one involving perceptual processing and one involving conceptual processing, prior to a blocked test phase to measure perceptual and conceptual priming. This allowed comparison of age effects in the following conditions: perceptual encoding, perceptual test [PP]; conceptual encoding, perceptual test [CP]; perceptual encoding, conceptual test [PC]; conceptual encoding, conceptual test [CC]. Priming tasks were matched on all characteristics except processing, making them highly comparable. The perceptual task employed the continuous identification (CID) paradigm (e.g., Stark & McClelland, 2000), in which participants made speeded identifications of words (studied/new) as they gradually emerged, and the conceptual task used a CV paradigm (e.g., Light et al., 2000), in which participants made speeded judgements as to whether words (studied/new) matched given category labels. Both tasks involved a speeded measure with response times (RTs) as the dependent variable, with all aspects matched as closely as possible (i.e., within-trial events and durations, see section “Procedure”).

A few other points should be noted: (1) Target items were presented in both the CID (perceptual) and CV (conceptual) tasks, meaning there was encoding-test perceptual overlap in both and only the type of processing differed. This approach was favoured over a conceptual task that does not present target items, such as CEG, to match tasks as far as possible and rule out age differences not attributed to processing (Craik et al., 1987; Kahana et al., 2002; Kahana & Wingfield, 2000). It was deemed that presenting targets should not distort priming in the CV task because, as reviewed above, perceptual stimulus features do not support conceptual priming. (2) It would be theoretically interesting to compare priming in the present conditions to an explicit recognition measure. Some prior studies have examined age effects by concurrently assessing priming and recognition trial-by-trial (for a recent example, see Ward et al., 2020). The concurrent method is notable because explicit memory and implicit memory for a given test item are captured within a few hundred milliseconds of one another, making them more suitable for comparison than when they are sampled in separate experimental phases involving a delay; however, a major limitation is that participants are aware that some items were previously studied. Although there is evidence that the CID task and similar speeded measures are unaffected by explicit contamination (e.g., Brown et al., 1991, 1996; MacLeod, 2008; Ward et al., 2013b), there is also evidence that explicit strategies can have a negative impact on conceptual priming in older adults (e.g., Mitchell & Perlmutter, 1986). Given this issue, and in light of the important effort to gain an accurate picture of implicit memory in ageing, no recognition assessment was incorporated in the present design.

## Hypotheses

**Figure 1.**
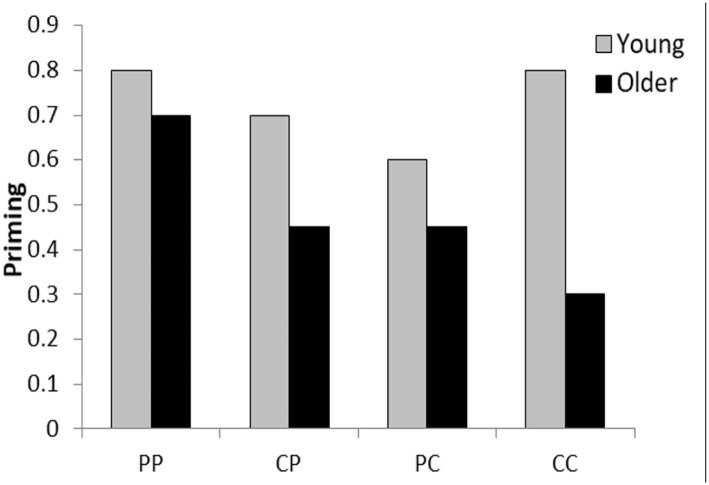
Predicted age differences in priming. PP: perceptual encoding, perceptual test; CP: conceptual encoding, perceptual test; PC: perceptual encoding, conceptual test; CC: conceptual encoding, conceptual test.

Hypotheses are numbered to correspond to the analyses in the “Results” section. In overview, it was anticipated that age differences in priming would vary across conditions, where CC > PC and CP > PP (Figure 1). In other words, the age difference in priming was expected to be greatest in the CC condition and smallest in the PP condition. This would be qualified by a main effect of Age (*Hypothesis 1*) and an Age × Processing (perceptual/conceptual encoding) × Test (CID/CV) interaction (*Hypothesis 2*). This was predicted on the basis of evidence that conceptual processing is affected to a greater extent by ageing than perceptual processing (e.g., Rybash, 1996), and encoding-test processing overlap yields greater priming than processing mismatch (e.g., Roediger & Blaxton, 1987a; Roediger & McDermott, 1993). Thus, on the perceptual test, greater priming was expected following perceptual than conceptual encoding in both age groups (PP > CP) (*Hypothesis 3*), while on the conceptual test greater priming was expected following conceptual than perceptual encoding in young adults (CC > PC), and vice versa in older adults (PC > CC) (*Hypothesis 4*).

## Method

### Participants and design

The task was constructed using Gorilla Experiment Builder (gorilla.sc) (see section “Procedure”), and all participants completed the experiment online using a desktop or laptop computer. Young participants (aged between 18 and 30 years) were recruited through Middlesex University subject pool and Prolific, and older participants (aged 65 years and above) through an existing partnership with the University of the Third Age (see Table 1 for participant characteristics). Participants were rewarded with course credit or Amazon e-vouchers at a rate of £8 per hour. Ethical approval was granted by Middlesex University Research Ethics Committee.

**Table 1.** Participant characteristics.

	Young adults *M* (*SD*)	Older adults *M* (*SD*)
Age (years)	21.69 (3.33)	73.23 (6.30)
*N* Male / Female	12 / 36	17 / 31
Education (years)^a^	15.42 (1.91)	16.71 (2.32)
Self-rated health (*N*)		
*Excellent*	26	7
*Good*	19	38
*Adequate*	3	3
*Poor*	0	0
*Very poor*	0	0
Mill Hill Vocabulary^a^	16.79 (4.63)	25.11 (4.06)
Early Dementia Questionnaire (EDQ)	–	3.94 (1.83)

*Note*. Mill Hill Vocabulary score (maximum 33) is based on the multiple-choice component of the Mill Hill Vocabulary Test (Raven et al., 1988), as a standard measure of pre-morbid intelligence, and the EDQ (Arabi et al., 2013) is a brief cognitive screening suitable for self-administration (see section “Procedure”).

a Significant difference between groups, *p* < .05.

Processing (perceptual/conceptual) during encoding and test was manipulated within participants in a counterbalanced blocked design. The four conditions included PP, CP, PC, and CC. An a priori power analysis (G*Power) was conducted to calculate the required sample size using an estimated effect size (*f* = 0.15) and alpha at .05. The non-sphericity correction was set to maximum (1/*m*–1, where *m* signifies the number of measurements), and a moderate correlation between measures was assumed (*r* = .5). In total, 96 participants (48 young and 48 older) were required, with an actual power of 0.945, ensuring an equal number of participants in the counterbalance rotations. Additional participants were only tested to replace any who did not meet the inclusion criteria, failed to complete the whole experiment, or failed to follow task instructions (see section “Results”).

There was no upper age limit for older adults, but it was a requirement that they be free of cognitive impairment and dementia. Older participants who expressed an interest in taking part first completed the Early Dementia Questionnaire (EDQ; Arabi et al., 2013) electronically, and only participants deemed free of dementia were invited to complete the online experiment (see section “Procedure”). It was also a requirement that all participants were fluent in English and had normal or corrected vision. Participants were required to tick a box at the start of the online experiment to provide consent and confirm that they met the eligibility criteria. Other background information collected included details of age, sex, years of education, self-rated health, and pre-morbid intelligence (Mill Hill Vocabulary Test, Raven et al., 1988).

### Stimuli

Stimuli included 176 concrete nouns from 20 taxonomic categories selected from the updated category norms by Van Overschelde et al. (2004). Words of medium frequency were selected (e.g., “hawk,” “chisel”), with number of letters ranging from 3 to 11 (*M* = 5.55) and number of syllables ranging from 1 to 4 (*M* = 1.70). One hundred and sixty words served as experimental items, arranged into eight lists of 20 items, with 2 items per category per list. Half of the experimental items represented living objects and half non-living objects. In total, 88 words were presented during encoding, that is, two lists in the perceptual block and two in the conceptual block, along with two filler items at the start and end of each block. The eight filler items were from different categories to experimental items and not presented elsewhere in the experiment. One hundred and sixty items were presented at test—80 per block (40 studied [20 from each encoding block] and 40 new). Lists were counterbalanced between participants such that each appeared an equal number of times in the perceptual and conceptual blocks and as studied (old) and new type.

### Procedure

Participants were asked to complete the online experiment in a quiet, private space. A link launched the experiment, which was compatible with any browser and operating system, but set up to prohibit the use of tablets and mobile phones. Experimental research is increasingly being conducted online and provides an excellent way of accessing large samples, especially at a time when in-person testing presents risks in relation to the Covid-19 pandemic. The Gorilla platform has demonstrated capability of running sensitive reaction time experiments and has replicated effects from well-known paradigms such as the flanker task (Anwyl-Irvine et al., 2019). Precise instructions were generated for participants both in terms of how to perform each stage of the experiment and concerning the conditions under which they should attempt the experiment. These included: (1) completing the experiment in a private space, free of distractions such as phones and television; (2) completing the experiment when they have ample time (the total duration was 35–40 min, including the experimental task [25–30 min] and background measures [10 min]) as it cannot be paused or restarted; and (3) reading all instructions thoroughly and only progressing to the experimental trials when they are confident that they understand and have completed the practice. Nevertheless, it is recognised that the environment can never be as controlled as a laboratory setting, and for this reason, close screening of the data was applied (see section “Results”).

#### Experimental task

**Figure 2.**
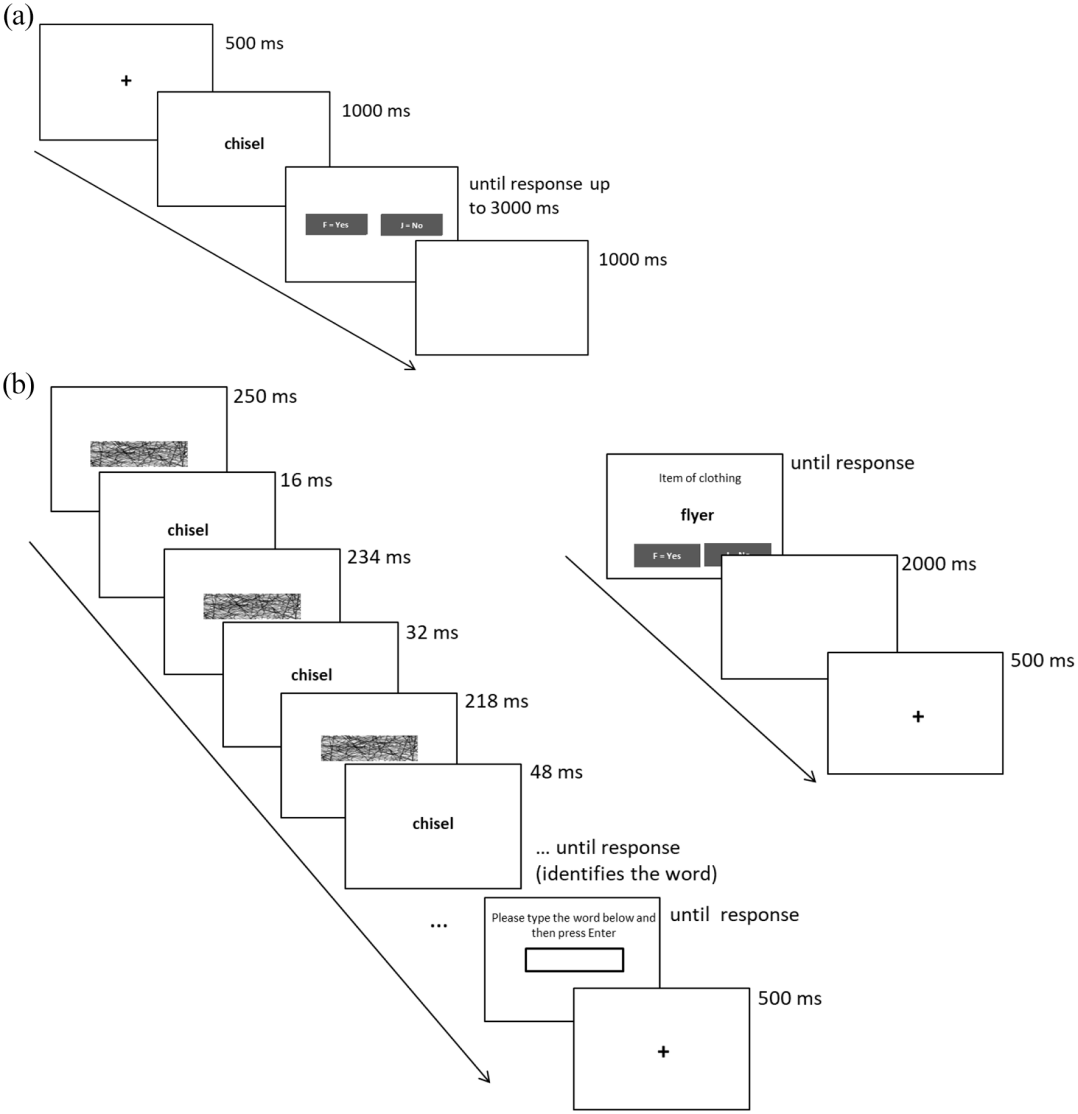
(a) Events in a single trial in the encoding phase. (b) Events in a single trial in the CID task (left) and CV task (right).

Participants were informed that the experiment consisted of separate phases, but they were not informed that stimuli from initial stages were repeated later. In the encoding phase, participants were presented with 88 words, one at a time, in black Open Sans lowercase text in the centre of a white background screen. The default size on Gorilla is 36 pixels, but as screen resolution varies between participants, items are automatically scaled to ensure that they fit within the confines of the zone. On each trial, a black fixation cross was presented for 500 ms, followed by a single word for 1,000 ms and a response screen for 3,000 ms (Figure 2). The 88 trials were separated into two counterbalanced blocks, one requiring perceptual processing and the other requiring conceptual processing. Each block contained 40 experimental trials presented in a new random order for each participant and 2 filler trials at the start and end. Blocks were favoured rather than intermixed trials to avoid processing overlap or interference between conditions, and participants were not informed in advance of the separate blocks. As such, they received instructions relevant to the first block at the start and completed five practice trials, and new instructions were presented prior to the second block.

In the perceptual block, participants judged whether the first and last letter of the word were in alphabetical order, and in the conceptual block, participants judged whether the item was living or non-living. The judgement in the perceptual block engaged participants with purely perceptual detail about the word, whereas judgements in the conceptual block required more elaborate appraisal of the item to decide whether it is living or non-living. Both decisions are objective and required a yes/no judgement. During the response screen, options “yes” and “no” were presented and participants had a maximum of 3,000 ms to respond using the F and J keyboard keys. RT was captured upon button press. Once pressed, a blank screen was presented for 1,000 ms prior to the next trial. If no response was made within 3,000 ms, the trial was discarded.

Following encoding, there was an interval of approximately 3 min while participants received instructions for the test phase and practised the initial priming segment. The test phase also comprised two counterbalanced blocks—the CID task (perceptual processing) and the CV task (conceptual processing), and the first block performed matched the first encoding block (perceptual/conceptual). Each block included 80 experimental trials—40 old words (20 from the perceptual and 20 from the conceptual encoding block) and 40 new words, presented in a new random order for each participant, plus 2 filler trials at the start and end. On each trial in the CID block, participants performed a speeded perceptual identification of a word, and on each trial in the CV block, participants performed a speeded CV (Figure 2).

In the CID task, participants were informed that their task was to identify words as quickly as possible. They were told that the words would initially be difficult to make out, but would appear to gradually emerge. Speed was emphasised, but participants were asked to try to be as accurate as possible. On each trial, a mask was presented for 250 ms, followed by a word for 16 ms (rate of one screen refresh) and the mask again for 234 ms, forming a 250-ms block. The block presentations continued with the word duration increasing by 16 ms on each alternate cycle and the mask duration decreasing by the same amount, with the result that the word appears to gradually emerge. RT was captured upon spacebar press, at which point the word disappeared and participants were prompted to type it into a box on the screen. If the spacebar was not pressed by 7,500 ms (i.e., when the word was fully displayed), the trial was discarded. Participants were prompted to press enter when they had finished typing the word to initiate the next trial. A fixation cross was presented for 500 ms prior to the next trial.

In the CV task, participants were informed that their task was to decide whether or not a word matches a given category as quickly as possible on each trial. As with the CID task, speed was emphasised, but participants were asked to try to be as accurate as possible. On each trial, a word was presented, along with a category label (e.g., “item of clothing”), and participants judged whether the word matched the category by selecting “yes” or “no” using the F and J keyboard keys. For half of the words, the correct category was presented, and for the other half, an incorrect category was presented, meaning that the correct response was “yes” for half of all trials. The word and response cue remained on the screen until response (RT captured), at which point the category and prompt disappeared. For consistency with the CID task, if no response was made by 7,500 ms the trial was discarded. To match the timings in the CID task as far as possible, after response a blank screen was presented for 2,000 ms, followed by a fixation cross for 500 ms prior to the next trial.

Immediately after the test phase, participants completed a short awareness questionnaire. Similar to that developed by Bowers and Schacter (1990), this included the following items: (1) *What do you think was the purpose of the identification and category selection tasks you just performed?* (2) *Did you notice anything about these tasks?* If participants failed to specify that some words were previously studied, they were classified as unaware and not required to complete the rest of the questionnaire. If they noticed that some words were previously studied, they were asked: (3) *Were you aware that some words had been shown previously in the experiment as you were performing the tasks, or did you become aware of this afterwards/in hindsight?* (4) *Did you suspect prior to the start of the second phase of the experiment that you would be tested on your memory of the words shown in the first phase?* (5) *Did you try to use your memory of the words to help you in the tasks in the second phase?* (6) *If yes, do you think this strategy helped you, and how so?* Participants who stated they became aware in hindsight were classified as unaware at the time of testing, and all other participants were classed as aware.

#### Background measures

The EDQ was completed by older participants prior to inviting them to perform the experimental task. This is a brief (5–10 min) cognitive screening appropriate for self-administration and was distributed to participants electronically. The questionnaire consists of 20 statements that participants must respond to on a Likert-type scale taking into account the previous 2 years, for example, “require checklist as memory support” “never (0),” “seldom (1),” “sometimes (2),” “always (3).” The numbers in parenthesis are summed to provide an overall score (range, 0–60), and a score of 8 or more indicates possible early dementia. Only participants scoring ⩽7 were invited to take part in the experiment. The first part of the online task collected background information from all participants, including age, sex, years of education, and self-rated health (5-point scale ranging from *excellent* to *very poor*), and upon completion of the experimental task, participants performed the multiple-choice part of the Mill Hill Vocabulary Test. This involved selecting an acronym from a choice of 6 to best describe a target word. This test includes 33 items in total, and each correct response is given a score of 1 (range, 0–33) (see Table 1 for summary scores).

## Results

The experiment was pre-registered on the Open Science Framework (osf.io/anfby). All steps and analyses were as per the pre-registered protocol. Participants were excluded if it was apparent that they did not meet the eligibility criteria, if they failed to complete the experimental task in full or follow instructions, or if they did not meet performance thresholds specified below. In total, there were 45 exclusions (19 young and 26 older adults), and additional participants were tested to achieve the required sample size of 96 usable participants. Ten exclusions (3 young and 7 older adults) were due to missing data (i.e., failure to complete the full experiment), and 35 (16 young and 19 older adults) were due to performance thresholds not being met (detailed in the relevant sections below). Analyses on the final sample were conducted using JASP (Version 0.9.2), using an alpha level of .05. All pre-registered analyses are reported, with partial eta squared for analysis of variance (ANOVA) effects and Cohen’s *d* for *t*-tests. For nonsignificant effects, Bayes Factor analysis (also using JASP) was conducted and BF10 values of less than 1/3 were considered support for the null hypothesis (Dienes, 2014). Raw data and analysis files for the final sample are available here: https://osf.io/z7vua/

### Encoding

The proportion of discarded trials due to time-out was low (1.9% and 1.6% for young and older adults, respectively). Mean accuracy (letter judgements in the perceptual block, and living/non-living judgements in the conceptual block) and RTs (for correct trials) can be found in Table 2. Fourteen young and 12 older participants with insufficient accuracy (<80% correct) were excluded. The 2 (Age) × 2 (Processing) ANOVA revealed main effects of Processing, *F*(1, 94) = 32.29, *p* < .001, $\eta_{p}^{2}=.256$ , and Age, *F*(1, 94) = 26.08, *p* < .001, $\eta_{p}^{2}=.217$ , on accuracy and no interaction, *F*(1, 94) = 0.77, *p* = .383, $\eta_{p}^{2}=.008$ (BF10 = 0.299). Performance was greater in the conceptual than the perceptual block and in older than in younger adults. There was a main effect of Processing on RT, *F*(1, 94) = 106.17, *p* < .001, $\eta_{p}^{2}=.530$ , and a Processing × Age interaction, *F*(1, 94) = 33.99, *p* < .001, $\eta_{p}^{2}=266$ , but no main effect of Age, *F*(1, 94) = 0.07, *p* = .787, $\eta_{p}^{2}=001$ (BF10 = 0.181), indicating significantly slower responding in the perceptual than the conceptual block in young adults.

**Table 2.** Performance in the detection task.

	Young *M* (*SD*)	Older *M* (*SD*)
Accuracy (%)		
Perceptual block	84.68 (9.31)	90.95 (6.92)
Conceptual block	91.53 (6.78)	95.97 (5.46)
RT (ms)		
Perceptual block	784 (294)	631 (239)
Conceptual block	388 (132)	521 (163)

*SD*: standard deviation; RT: response time.

### Priming

For each participant, trials with RTs <200 ms or >3 *SD*s from the mean were trimmed. Trials associated with incorrect identifications were removed (i.e., incorrect word typed, minor spelling mistakes permitted in the CID task, and incorrect category selected in the CV task). Two young and seven older adults with insufficient accuracy (>20% incorrect) were excluded. In the CV task, RTs to be studied and new trials necessitating a “yes” response (i.e., word paired with the correct category) or a “no” response (i.e., word paired with an incorrect category) were collapsed. Priming in each condition was calculated by subtracting each participant’s mean old-item RT from their mean new-item RT, expressed in proportion to their mean baseline (new-item) RT: (RTnew−RTold)/RTnew. Priming proportional to baseline was favoured because slower responding in older than young adults can artificially elevate priming when RT difference scores are used (e.g., Faust et al., 1999).^1^ For raw RTs, see Table 3.

**Table 3.** RTs (ms) in young and older adults in the CID and CV priming tasks.

	Young *M* (*SD*)	Older *M* (*SD*)
CID		
Perceptual (PP)	1,759 (699)	1,985 (751)
Conceptual (CP)	1,791 (663)	1,904 (740)
New	1,912 (676)	2,040 (752)
CV		
Perceptual (PC)	1,207 (320)	1,384 (225)
Conceptual (CC)	1,230 (324)	1,385 (222)
New	1,273 (393)	1,388 (218)

*SD*: standard deviation; CID: continuous identification; PP: perceptual encoding, perceptual test; CP: conceptual encoding, perceptual test; CV: category verification; PC: perceptual encoding, conceptual test; CC: conceptual encoding, conceptual test.

**Figure 3.**
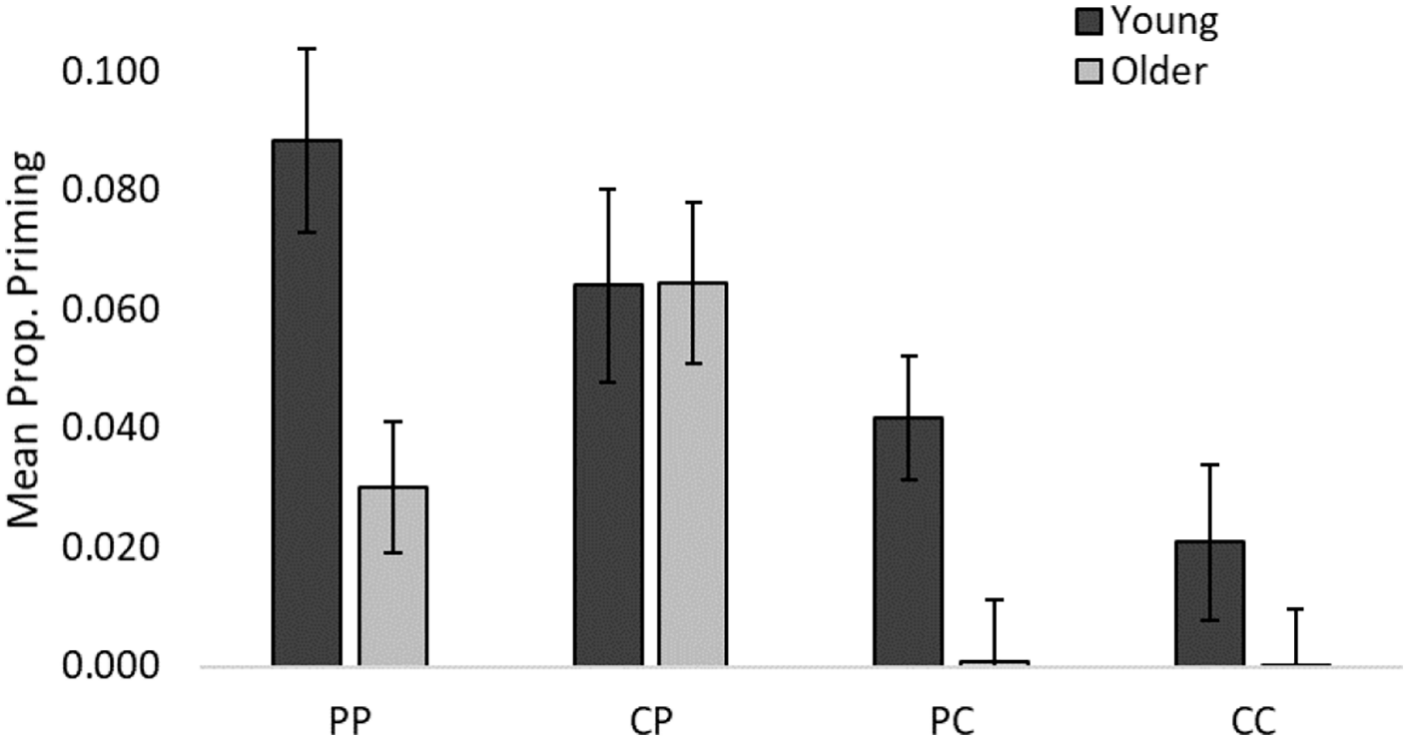
Priming in young and older adults in the PP, CP, PC, and CC conditions Standard error bars.

Priming (Figure 3) in young adults was significantly above zero in all conditions apart from CC—PP: *t*(47) = 5.75, *p* < .001, *d* = 0.83; CP: *t*(47) = 3.86, *p* < .001, *d* = 0.56; PC: *t*(47) = 3.98, *p* < .001, *d* = 0.57; CC: *t*(47) = 1.59, *p* = .118, *d* = 0.23 (BF10 = 0.508), but was only significantly above zero in older adults in the PP and CP conditions—PP: *t*(47) = 2.67, *p* = .010, *d* = 0.39; CP: *t*(47) = 4.73, *p* < .001, *d* *=* 0.68; PC: *t*(47) = 0.06, *p* = .951, *d* = 0.01 (BF10 = 0.157); CC: *t*(47) = 0.05, *p* = .965, *d* = 0.01 (BF10 = 0.157) (all two-tailed). The 2 (Age) × 2 (Processing) × 2 (Test) ANOVA revealed a main effect of Age, *F*(1, 94) = 9.83, *p* = .002, $\eta_{p}^{2}=.095$ (*Hypothesis 1*), and a main effect of Test, *F*(1, 94) = 14.52, *p* < .001, $\eta_{p}^{2}=.134$ , but there was no main effect of Processing, *F*(1, 94) = 0.22, *p* = .644, $\eta_{p}^{2}=.002$ (BF10 = 0.118), and the expected Age × Processing × Test interaction (*Hypothesis 2*) was not significant, *F*(1, 94) = 2.19, *p* = .143, $\eta_{p}^{2}=.023$ (BF10 = 0.359). There was a significant Age × Processing interaction, *F*(1, 94) = 10.01, *p* = .002, $\eta_{p}^{2}=.096$ , but no other interactions—Age × Test: *F*(1, 94) = 0.009, *p* = .924, $\eta_{p}^{2}<.001$ (BF10 = 0.077); Processing × Test: *F*(1, 94) = 1.34, *p* = .250, $\eta_{p}^{2}=.014$ (BF10 = 0.113).

Years of education, Mill Hill vocabulary scores (Table 1), and encoding phase accuracy significantly differed between groups, so as per the pre-registered protocol the ANOVA was repeated with the relevant variables entered as covariates. Upon checking assumptions, it was found that years of education and Mill Hill vocabulary scores were highly correlated, *r*(94) = .27, *p* = .009, as were Mill Hill scores and encoding phase accuracy in the perceptual block, *r*(94) = .35, *p* = .001, and conceptual block, *r*(94) = .22, *p* = .034. Therefore, years of education and encoding phase accuracy in the perceptual and conceptual blocks were entered into the model as covariates, and Mill Hill vocabulary scores were not included. The analysis of covariance (ANCOVA) revealed a main effect of Age, *F*(1, 91) = 4.90, *p* = .029, $\eta_{p}^{2}=.051$ , a main effect of Test, *F*(1, 91) = 4.37, *p* = .039, $\eta_{p}^{2}=.046$ , and an Age × Processing interaction, *F*(1, 91) = 7.95, *p* = .006, $\eta_{p}^{2}=.080$ .

The post-test awareness questionnaire revealed that 17 young and 9 older adults were aware during the experiment that items from the encoding phase were repeated in later phases (Table 4). The pre-registered threshold for statistical comparison of priming in aware and unaware participants was 20% aware participants per group. Therefore, the 2 (Processing) × 2 (Test) × 2 (Aware) ANOVA was performed on young adults. Awareness did not affect priming or interact with manipulated factors: The main effect of Awareness was not statistically reliable, *F*(1, 46) = 3.06, *p* = .087, $\eta_{p}^{2}=.062$ (BF10 = 0.524), there was no interaction with Processing, *F*(1, 46) = 0.29, *p* = .593, $\eta_{p}^{2}=.006$ (BF10 = 0.230), no interaction with Test, *F*(1, 46) = 1.76, *p* = .191, $\eta_{p}^{2}=.037$ (BF10 = 0.214), and no Processing × Test × Awareness interaction, *F*(1, 46) = 0.001, *p* = .975, $\eta_{p}^{2}<.001$ (BF10 = 0.287).

**Table 4.** Priming in aware and unaware participants.

	Young *M* (*SD*)	Older *M* (*SD*)
Aware		
PP	0.13 (0.10)	0.05 (0.07)
CP	0.09 (0.13)	0.09 (0.07)
PC	0.45 (0.09)	0.04 (0.05)
CC	0.02 (0.10)	< 0.01 (0.07)
Unaware		
PP	0.07 (0.10)	0.02 (0.08)
CP	0.05 (0.10)	0.06 (0.10)
PC	0.04 (0.06)	–0.01 (0.07)
CC	0.02 (0.09)	< 0.01 (0.06)

*SD*: standard deviation; PP: perceptual encoding, perceptual test; CP: conceptual encoding, perceptual test; PC: perceptual encoding, conceptual test; CC: conceptual encoding, conceptual test.

### Exploratory analyses

The main analysis revealed effects of Age, Test, and an Age × Processing interaction. Priming was generally greater in young than older adults and on the perceptual (CID) than the conceptual (CV) priming task. However, follow-up tests for the interaction confirmed significant age differences in priming following perceptual encoding (PP and PC) but not conceptual encoding (CP and CC), with the largest effect size for the age difference in the PP condition—PP: *t*(94) = 3.14, *p* = .002, *d* = 0.64, PC: *t*(94) = 2.81, *p* = .006, *d* = 0.57; CP: *t*(94) = 0.05, *p* = .961, *d* = 0.01 (BF10 = 0.215), CC: *t*(94) = 1.33, *p* = .188, *d* = 0.27 (BF10 = 0.466) (all two-tailed independent *t*-tests; Bonferroni-corrected *p* = .013).

Although the expected Age × Processing × Test interaction was not significant, the observed BF10 value greater than 1/3 does not provide strong support in favour of the null hypothesis of no interaction. Interestingly, priming in young adults was significantly greater on the perceptual than the conceptual test only when encoding was perceptual—that is, PP > PC, *t*(47) = 2.42, *p* = .019, *d* = 0.35; CP = CC, *t*(47) = 1.71, *p* = .094, *d* = 0.25, BF10 = 0.603, but the opposite was true in older adults—their priming was significantly greater on the perceptual than the conceptual test only following conceptual encoding—that is, CP > CC, *t*(47) = 3.83, *p* < .001, *d* = 0.55; PP = PC, *t*(47) = 1.80, *p* = .078, *d* = 0.26, BF10 = 0.695 (two-tailed paired *t*-tests; Bonferroni-corrected *p* = .025). However, it should be noted that the BF10 values greater than 1/3 do not provide strong support for the null hypothesis of no difference between these comparison conditions.

## Discussion

Processing requirements have varied considerably between studies examining age effects on priming, and this may have contributed to the mixed outcomes. Put simply, if ageing does not equally affect the ability to engage in perceptual and conceptual processing, this may explain why age differences in priming have emerged in some studies and not others. In an attempt to clarify this issue, this study systematically manipulated processing requirements (perceptual/conceptual) at encoding prior to matched perceptual and conceptual priming tasks. Main effects of Age and Task, and an Age × Processing interaction emerged, demonstrating reliably greater priming in young than older adults when encoding was perceptual—that is, on the PP (perceptual encoding, perceptual test) and PC (perceptual encoding, conceptual test) conditions, and equivalent priming in the CP (conceptual encoding, perceptual test) and CC (conceptual encoding, conceptual test) conditions.

To be able to unpack age differences in priming as a function of processing characteristics, it is important that tasks are appropriately classified. The continuous identification (CID—perceptual) and category verification (CV—conceptual) tasks were selected based on their established processing requirements and matched as closely as possible on all characteristics except processing. Both captured an RT measure, and all within-trial events and durations were aligned (see sections “Introduction” and “Method”). Priming was generally lower on the conceptual than the perceptual task, but age effects did not interact with the type of task, pointing to a decline in both perceptual and conceptual priming with age. The findings are consistent with several prior studies reporting reliable age-related reductions in perceptual priming (e.g., Abbenhuis et al., 1990; Chiarello & Hoyer, 1988; Keane et al., 2004; Russo & Parkin, 1993; Small et al., 1995; Soldan et al., 2009; Ward et al., 2013b, 2015, 2020; Wiggs & Martin, 1994), and conceptual priming (e.g., Grober et al., 1992; Jelicic et al., 1996; Light et al., 2000; Maki et al., 1999; Maki & Knopman, 1996; Stuart et al., 2006). Many of these studies used firmly established perceptual/conceptual tasks (e.g., word-fragment completion, picture-fragment identification, CEG), but some used WSC, which, although largely considered a perceptual test, has been the subject of debate where processing requirements are concerned (discussed in Mitchell & Bruss, 2003; see also Ryan et al., 2001; Toth, 2000).

To the best of this author’s knowledge, all adequately powered studies using the CID task have uncovered a reliable reduction with age (recently reviewed in Ward & Shanks, 2018; note that there are instances of age-invariant CID priming in amnesia, for example, Conroy et al., 2005). This task and other such speeded perceptual measures are considered relatively “clean” tests. The clear goal to identify items as quickly as possible does not generally allow different strategies between participants, meaning lower response variability in comparison with other tasks like WSC in which the goal is less rigid and participants have more time. This issue was discussed in detail by Buchner and Wippich (2000), who demonstrated greater statistical reliability of a speeded perceptual identification task compared with WSC and greater sensitivity to age differences. There is also evidence that perceptual identification tasks including the CID task used in this study are unaffected by explicit contamination (e.g., Brown et al., 1996; Ward et al., 2013b). However, relatively few studies have employed the CV task. This was deemed most appropriate in the present study over other popular conceptual tasks such as CEG, because it is based on an RT measure and allowed closer procedural matching to the perceptual task. As discussed by Light et al. (2000), this task is inherently conceptual because deciding whether a word is an instance of a particular category requires access to meaning. They reported lower CV priming in older than young adults in their Experiment 2, but no reliable age difference in Experiment 3 (note that these experiments involved an attentional manipulation). They also reported similar priming in young and older adults on a CEG task (Experiment 1). In their Experiment 4, involving only young adults, they observed no effect on CV priming of varying processing at encoding (pleasantness ratings vs syllable counting). In their Experiment 2, priming did not reach significance in older adults (.006), similar to the present CV task (<.001, collapsed across perceptual/conceptual encoding), but older adults’ priming was greater in Experiment 3 (.055) when RTs were reduced by presenting the category label prior to the target word on each trial. It is also worth noting that CV priming was lower in young adults in the present study (.031, collapsed across perceptual/conceptual encoding) compared with Light et al. (.066 in Experiment 2 and .072 in Experiment 3), which could have reduced the strength of the observed age difference.

A few other differences between outcomes on the CID and CV should be noted. First, more CV trials were lost for older adults (8.83%) than young adults (5.83%) due to time-out and inaccuracies, while the level of discarded trials in the perceptual task was equivalent in young and older adults (4.06% and 4.84%, respectively). Second, RTs were slower in the CID than the CV task in both groups, no doubt reflecting the fact that words were gradually clarified in the CID task but were fully presented in the CV task. This is unlikely to have distorted the age effect because the slowing was observed in both age groups and did not disproportionately affect baseline (i.e., new-item) RTs. Furthermore, priming scores proportional to baseline were calculated. Finally, 17 young and 9 older participants were aware during testing that items from encoding were represented. More young than older adults may therefore have attempted to use an explicit strategy in the CID and/or CV tasks. Although analysis revealed no statistically significant difference in priming between aware and unaware young participants, priming was numerically greater in aware participants and the BF10 value of 0.524 does not provide strong evidence of there being no difference.

The present data suggest that age effects on priming are dependent upon the manner of encoding. Priming was greater in young than older adults only following perceptual encoding—that is, in the PP and PC conditions. Of particular note, the largest age difference was observed in the PP condition while priming was equivalent in the CP condition, both involving an identical priming task. Thus, the type of encoding was responsible for the emergence or elimination of a reliable age difference. Given evidence that older adults are impaired in semantic encoding (e.g., Eysenck, 1974; Morcom et al., 2003; Morcom & Rugg, 2004; see also Ward et al., 2017), it was predicted that age differences in priming would be larger when encoding engages participants with semantic features of stimuli, where it was reasoned that young adults would be at a processing advantage. However, older adults outperformed young adults at encoding and there was no evidence of semantic impairment. It is worth noting, though, that the Age × Processing interaction remained significant when age differences in encoding phase accuracy were partialled out. Every effort was made to ensure qualitatively equivalent processing in young and older adults during the encoding phase; however, young adults appeared to find the perceptual block more challenging—both groups were less accurate in the perceptual than the conceptual block, but young adults alone were significantly slower in this block. It is possible, although unlikely, that young adults engaged in additional processing in the perceptual encoding block that could have heightened their priming in the PP and PC conditions, exaggerating the age difference. One would generally expect faster responses for perceptual judgements than those that require elaborate semantic processing, but the present slower responding in the perceptual block likely reflects that participants needed to appraise two independent features of each word—the first and last letter—to decide if they were in alphabetical order. Although a purely perceptual judgement, this involved additional time than when single items were appraised in the conceptual block to make living/non-living judgements.

No prior study has fully crossed processing requirements at encoding and test to examine interactions with age effects on priming. However, the present PP and CC conditions are similar to those in Stuart et al. (2006), in which participants performed a perceptual priming task following perceptual encoding or a conceptual priming task following conceptual encoding. In their study, priming was reduced by age in the conceptual but not the perceptual condition; however, (1) it is unknown whether this was driven by the type of processing at encoding or type of test as there was no examination of conceptual priming following perceptual encoding, or vice versa, and (2) a between-subjects design was used with WSC as the perceptual test and CEG as the conceptual test, neither of which can be directly compared and both of which have been subject to criticism. More recently, Ward et al. (2020) examined priming for attended and unattended objects on a CID (perceptual) priming task following either perceptual (shape judgements) or conceptual (natural/manufactured judgements) encoding, yielding conditions similar to the present PP and CP conditions. Priming (for attended items) was reduced by age, but in contrast to the present study there was no interaction with encoding. Comparisons should be made with caution, however, due to key differences between studies. Ward et al. manipulated perceptual/conceptual encoding between participants, with a concurrent within-participants attention manipulation, but argued that the processing manipulation had been ineffective. Furthermore, there was no examination of conceptual priming, and the experiment involved comparison of five lifespan groups rather than simply young and older adults. The present study is the first to directly compare matched PP, PC, CP, and CC conditions, clarifying age effects on perceptual and conceptual priming as a function of encoding.

A final point concerns the interesting paired comparisons of priming across the perceptual and conceptual tasks. Young adults achieved greater priming on the perceptual than the conceptual priming task when encoding was perceptual (i.e., PP > PC and CP = CC), but the opposite was true of older adults—their priming was greater on the perceptual than the conceptual priming task following conceptual encoding (i.e., CP > CC and PP = PC). These observations concern exploratory analyses and should be interpreted with caution given the BF10 values greater than 1/3, but nevertheless are interesting and worthy of further investigation in future studies. High priming in young adults in the PP condition is predicted by accounts that specify a benefit of an overlap in processing characteristics between encoding and test (e.g., Roediger & Blaxton, 1987a; Roediger & McDermott, 1993)—in this case, perceptual encoding coupled with a perceptual test. However, this account would also predict (as was hypothesised) high priming in young adults in the CC condition—conceptual encoding coupled with conceptual test. Furthermore, the present data suggest that encoding-test processing overlap is not important for older adults’ priming—it was greatest on the perceptual test following conceptual encoding. It is interesting that no reliable priming effects were observed for older adults on the conceptual priming task (PC and CC conditions), nor for young adults in the CC condition. However, the CV task was sensitive to priming effects, observed in young adults when prior encoding was perceptual (i.e., the PC condition), so it appears that ageing and manner of processing during encoding are key factors in determining conceptual priming.

To conclude, this study set out to clarify age effects in priming by systematically manipulating processing requirements at encoding and test. There was an age-related decline in both perceptual and conceptual priming, significant only when encoding was perceptual in nature. Priming was lower in both age groups on the conceptual than the perceptual priming task. The observed age effects have important implications for our theoretical understanding of the structure of memory. Given well-documented declines in explicit memory with age, reports of spared priming have often been cited as key evidence for a functionally independent implicit memory system (e.g., Gabrieli, 1998, 1999; Mitchell, 1989; Mitchell et al., 1990; Schacter, 1987; Schacter & Tulving, 1994; Squire, 1994, 2004, 2009; Tulving & Schacter, 1990, but see Berry et al., 2006, 2008a, 2008b, 2012; Buchner & Wippich, 2000; Nosofsky et al., 2012). However, the observed decline in priming with age may present a challenge for such views.

## Notes

1 Analyses were based on priming calculated in proportional to baseline reaction times (RTs) given evidence that slower responding in older than young adults can artificially elevate priming when pure RT difference scores are used (e.g., Faust et al., 1999). Indeed, older adults were slower at baseline in the present study. However, there are also logical arguments in favour of using RT difference scores (e.g., see Manis et al., 1980; Mitchell, 1989), and one can find RT difference scores for the present study on the OSF here: https://osf.io/z7vua/
